# Impact of prior osteotomy and osteosynthesis on long-term outcomes after total hip arthroplasty: a 1:1 Mahalanobis distance-matched registry study of 5,392 patients

**DOI:** 10.1302/2633-1462.68.BJO-2025-0067

**Published:** 2025-08-11

**Authors:** Nele Wagener, Yinan Wu, Alexander Grimberg, Christian Hipfl, Sebastian Hardt

**Affiliations:** 1 Center for Musculoskeletal Surgery, Charité-University Medicine Berlin, Berlin, Germany; 2 EPRD Deutsches Endoprothesenregister gGmbH, Berlin, Germany

**Keywords:** Total hip arthroplasty (THA), Osteotomy, Osteosynthesis, Revision risk, Mortality, osteosyntheses, osteotomies, cemented femoral components, infections, periprosthetic fractures, cementless femoral components, Comorbidities, BMI, hip and knee arthroplasties

## Abstract

**Aims:**

Patients with a history of osteotomy or osteosynthesis pose distinct challenges in total hip arthroplasty (THA) due to altered anatomy and biomechanics. Although THA is an established intervention for degenerative hip disease, limited evidence exists on its long-term outcomes in this cohort, especially regarding revision rates, mortality, and complications. This registry study aimed to determine these outcomes using data from a large national registry.

**Methods:**

This registry study analyzed data from the German Arthroplasty Registry (EPRD), which captures approximately 70% of all hip arthroplasties in Germany. Among 418,409 patients undergoing THA between November 2012 and March 2024, 5,392 were included after 1:1 Mahalanobis distance matching for age, sex, BMI, and comorbidities: 2,696 patients with a history of osteotomy or osteosynthesis compared with 2,696 patients without. Kaplan-Meier survival curves estimated revision and mortality risks over an eight-year follow-up.

**Results:**

Over eight years, patients with prior osteotomy or osteosynthesis had significantly higher revision (6.8%, n = 183/2,696 vs 3.9%, n = 105/2,696, p = 0.002) and mortality (25.2%, n = 679/2,696 vs 20.4%, n = 550/2,696, p < 0.001) rates than those without prior hip surgery. Infection (17%, n = 22/131 vs 16%, n = 15/94), periprosthetic fracture (14%, n = 18/131 vs 12%, n = 11/94), and dislocation (14%, n = 18/131 vs 8.5%, n=8/94) were leading causes of revision. For cementless femoral components, prior-surgery patients had an eight-year revision rate of 7.3%, n = 143/1,957 compared with 3.6%, n = 71/1,958 (p = 0.003) and a mortality rate of 17.3%, n = 339/1,957 compared with 10.9%, n = 213/1,958 (p < 0.001). For cemented femoral components, revision rates were 4.9%, n = 36/739, compared with 4.7%, n = 35/738 (p = 0.330), and mortality 46.3%, n = 342/739, compared with 43.0%, n = 317/738 (p < 0.001). At one year, the revision rate in the prior-surgery group was already elevated at 3.7% (95% CI 3.1 to 4.5; n = 100/2,696) compared with 2.6% (95% CI 2.0 to 3.3; n = 70/2,696) in controls, diverging further over time.

**Conclusion:**

Patients with prior osteotomy or osteosynthesis undergoing THA face higher long-term revision and mortality risks, particularly with cementless stem fixation. Infection, periprosthetic fracture, and dislocation are key causes of revision.

Cite this article: *Bone Jt Open* 2025;6(8):915–923.

## Introduction

Total hip arthroplasty (THA) is a mainstay of orthopaedic treatment for end-stage hip pathologies, especially in patients who present with anatomically altered hip joints following osteotomies or osteosyntheses. Such surgical interventions, which are frequently performed to correct deformities like coxa vara, coxa valga, developmental dysplasia of the hip (DDH), or to stabilize fractures of the proximal femur and pelvis, are integral components of contemporary orthopaedic practice on a global scale.^1^ While osteotomies are often indicated in younger individuals to improve joint mechanics, osteosyntheses primarily focus on the stabilization of traumatic injuries.

Studies indicate that roughly 20% to 25% of patients who undergo osteotomies or osteosyntheses eventually progress to secondary osteoarthritis (OA), suggesting that mechanical or structural changes caused by these interventions may predispose patients to degenerative joint disease.^2^ Additionally, about 10% of hip OA cases are directly attributable to underlying DDH.^3,4^ In DDH, a shallow and insufficiently stable acetabulum fails to provide adequate femoral head coverage, resulting in altered load distribution and joint instability that can complicate THA procedures. These technical complexities often necessitate specialized approaches such as acetabular reconstruction or femoral realignment to achieve proper implant orientation, adequate joint congruency, and optimal biomechanical balance. Historically, patients with these challenging anatomies have experienced reduced implant survival rates and elevated revision risks, particularly in the early postoperative phase.^5-7^

Biomechanical alterations secondary to traumatically or surgically induced changes in hip anatomy contribute to secondary OA of the hip joint.^8^ Other contributing factors include congenital or acquired deformities and inflammatory conditions, highlighting the multifactorial aetiology of secondary OA when contrasted with the idiopathic nature of primary OA.^9^ Despite the recognized complexities of THA in this subgroup, the evidence base concerning long-term clinical outcomes, particularly revision rates, mortality risk, and the role of fixation techniques (cemented vs cementless), remains limited.

This study aims to fill that knowledge gap by examining the long-term results of THA in patients with a history of osteotomies or osteosyntheses compared to those with no prior hip surgeries. Special emphasis is placed on assessing the impact of femoral component fixation methods on revision and mortality rates, while also exploring other clinically relevant risk factors in these populations.

## Methods

### Data source

We used data from the German Arthroplasty Registry (Endoprothesenregister Deutschland, EPRD),^10^ covering the period from November 2012 to 30 March 2024. A total of 570,286 patients who underwent THA were documented, based on 1) billing records from statutory health insurers; 2) a product database maintained by implant manufacturers; and 3) electronic case reports from participating hospitals. Approximately 70% of all hip and knee arthroplasties in Germany are included, ensuring near-complete follow-up via linked insurance data. Coding followed Operation and Procedure Classification System (OPS)^11^ 301 and International Classification of Diseases of the World Health Organization (ICD)-10 guidelines.^12^ The EPRD has ethical approval from the University of Kiel (D 473/11).

### Patient selection and endpoints

**Fig. 1:**
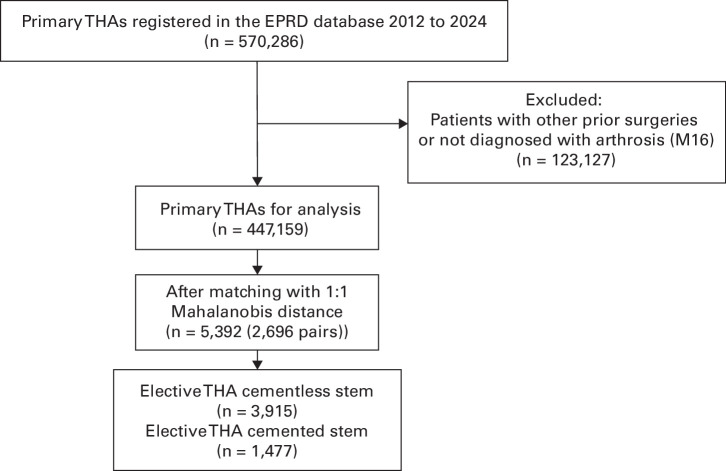
Patient flow of study population. EPRD, Endoprothesenregister Deutschland; THA, total hip arthroplasty.

Overall, 5,392 THAs met the inclusion criteria (Figure 1). Of these, 2,696 patients had a prior osteotomy or osteosynthesis (‘osteotomy + post-traumatic’), and the remaining 2,696 had osteoarthritis with no previous hip intervention (‘non-prior + OA’). Primary endpoints were THA survival (time to revision), revision rates, and mortality. Because implant failure and mortality compete, death was considered a competing risk.

Patients were assigned to two main cohorts (with or without previous osteotomy/osteosynthesis). Because the EPRD could only analyze osteotomy and osteosynthesis patients collectively, they were not evaluated as separate subgroups. To investigate femoral component fixation, we conducted a subgroup analysis that only included cemented compared with cementless components. Acetabular fixation was not considered, as our focus was solely on the femoral component. Comorbidities were captured using the modified Elixhauser Comorbidity Index (ECI).

### Patient characteristics

The following data are presented: the distribution of patients’ sex, age at admission, BMI categories, Elixhauser comorbidity scores, mortality rates, and implant type (cementless vs cemented) in patients with prior osteotomy or osteosynthesis compared to those without prior surgery. The chi-squared test and Mann-Whitney U test were used, as indicated.

After matching, both groups consisted of 2,696 patients, with comparable baseline characteristics (Table I). The mean age at admission was 66 years (SD 14) in each cohort, and the sex distribution was nearly identical with 1,425 females (53%) and 1,271 males (47%) in both groups (p > 0.900, chi-squared test). BMI categories were likewise similar between the two groups, with roughly 2% underweight, 30% normal weight, 28% pre‐obese, and around 12%, 4%, and 1% to 2% in the respective obese class I, II, and III ranges. The mean Elixhauser comorbidity score was 2.0 (SD 4.8) in both cohorts (p > 0.900). Notably, the mortality rate was higher in the group with prior osteotomy or osteosynthesis (12%) compared with those without such surgery (10%; p = 0.006). In contrast, the proportion of cementless (73%) compared with cemented (27%) femoral components did not differ significantly between groups (p > 0.900).

**Table I.** Descriptive statistics after matching.

Variable	Osteotomy/osteosynthesis (n = 2,696)^1^	Without prior surgery (n = 2,696)^1^	p-value
**Sex, n (%)**			> 0.900*
Female	1,425/2,696 (53)	1,425/2,696 (53)	
Male	1,271/2,696 (47)	1,271/2,696 (47)	
Mean age, yrs (SD)	66 (14)	66 (14)	> 0.900†
**BMI category, n (%)**			> 0.900*
Underweight (< 18.5 kg/m^2^)	56/2,696 (2.1)	56/2,696 (2.1)	
Normal (18.5 to 24.99 kg/m^2^)	804/2,696 (30)	804/2,696 (30)	
Pre-obese (25.0 to 29.99 kg/m^2^)	753/2,696 (28)	753/2,696 (28)	
Obese 1 (30.0 to 34.99 kg/m^2^)	314/2,696 (12)	314/2,696 (12)	
Obese 2 (35.0 to 39.99 kg/m^2^)	116/2,696 (4.3)	116/2,696 (4.3)	
Obese 3 (≥ 40 kg/m^2^)	37/2,696 (1.4)	37/2,696 (1.4)	
Missing	616/2,696 (23)	616/2,696 (23)	
Mean Elixhauser score (SD)	2.0 (4.8)	2.0 (4.8)	> 0.900†
**Mortality status, n (%)**			0.006*
Alive	2,361/2,696 (88)	2,425/2,696 (90)	
Dead	335/2,696 (12)	271/2,696 (10)	
**Subgroup, n (%)**			> 0.900*
Elective THA, cementless component	1,957/2,696 (73)	1,958/2,696 (73)	
Elective THA, cemented component	739/2,696 (27)	738/2,696 (27)	

* Chi-squared test.

† Mann-Whitney U test.

THA, total hip arthroplasty.

### Statistical analysis

All patients were adults undergoing elective THA. A 1:1 Mahalanobis matching controlled for confounding variables (age, sex, ECI, BMI, cement type).^13^ Descriptive statistics summarized baseline characteristics. Group comparisons employed chi-squared tests for categorial variables and independent-samples *t*-tests or Mann-Whitney U tests for continuous variables, depending on distribution. Kaplan-Meier analysis and log-rank tests assessed survival differences (significance at p < 0.05). All statistical analyses were conducted using R (version 4.2, R Foundation for Statistical Computing, Austria).

## Results

Table II provides an overview of the indications for revision surgery in patients with prior osteotomy or osteosynthesis (n = 131) compared with those without such a history (n = 94). Infection was the most frequently documented cause in both cohorts (n=22/131, 17% vs n=15/94, 16%), followed by periprosthetic fracture (n=18/131, 14% vs n=11/94, 12%) and dislocation (n=18/131, 14% vs n=8/94, 8.5%). Loosening of the cup occurred in n=10/131 (7.6%) of revisions among patients with prior surgery and n=5/94 (5.3%) in those without; loosening of the femoral component was noted in n=9/131 (6.9%) and n=4/94 (4.3%), respectively. Wear was relatively infrequent (n=2/131, 1.5% vs n=3/94, 3.2%), and malalignment accounted for n=5/131 (3.8%) compared with n=3/94 (3.2%) of cases. The analysis also revealed that other reasons were documented in n=14/131 (11%) vs n=12/94 (13%) of revisions, while missing data were present in n=33/131 (25%) and n=32/94 (34%) of cases, respectively.

**Table II.** Reasons for revision, presented as n (%).

Variable	Osteotomy and osteosynthesis (n = 131)	Without prior surgery (n = 94)
Infection	22 (17)	15 (16)
Loosening (cup)	10 (7.6)	5 (5.3)
Loosening (femoral component)	9 (6.9)	4 (4.3)
Loosening (cup and femoral component)	0 (0)	1 (1.1)
Osteolysis with fixed component (cup)	0 (0)	0 (0)
Osteolysis with fixed component (femoral component)	0 (0)	0 (0)
Osteolysis with fixed component (cup and femoral component)	0 (0)	0 (0)
Periprosthetic fracture	18 (14)	11 (12)
Dislocation	18 (14)	8 (8.5)
Wear	2 (1.5)	3 (3.2)
Component failure	0 (0)	0 (0)
Malalignment	5 (3.8)	3 (3.2)
Progression of arthrosis	0 (0)	0 (0)
Condition after removal	0 (0)	0 (0)
Other reasons	14 (11)	12 (13)
Missing	33 (25)	32 (34)

**Fig. 2:**
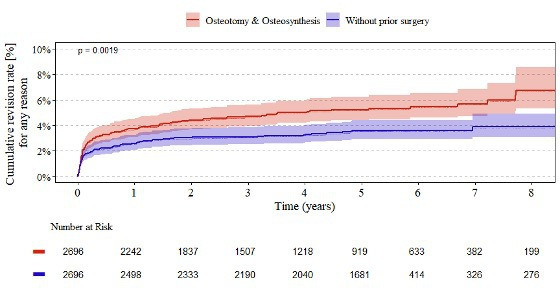
Endpoint revision of all total hip arthroplasties. Kaplan–Meier estimates of cumulative revision rates at one to eight years following total hip arthroplasty in patients with and without prior osteotomy or osteosynthesis. 95% CIs and numbers at risk are shown below the figure.

Figure 2 depicts the cumulative revision rates over an eight-year follow-up period. At one year, patients with prior osteotomy or osteosynthesis had a revision rate of 3.7% (95% CI 3.1 to 4.5), compared with 2.6% (95% CI 2.0 to 3.3) in those without prior surgery. The disparity in revision rates between these two groups widened over time, reaching 6.8% (95% CI 5.3 to 8.6) compared with 3.9% (95% CI 3.1 to 4.9) at eight years. The overall difference between the two cohorts was statistically significant (p = 0.0019).

**Fig. 3:**
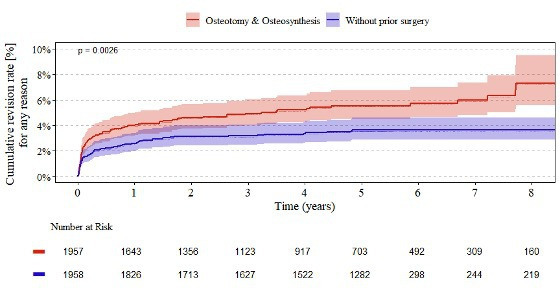
Endpoint revision of uncemented femoral components. Kaplan–Meier estimates of the cumulative revision rate for total hip arthroplasty in patients with previous osteotomy and osteosynthesis compared to those without prior surgery, reported annually up to eight years postoperatively. The estimates are accompanied by their respective 95% CIs.

Figure 3 illustrates the revision rates for uncemented femoral components up to eight years postoperatively. At one year, the cumulative revision rate in patients with a history of osteotomy or osteosynthesis was 4.0% (95% CI 3.2 to 5.0), compared to 2.6% (95% CI 2.0 to 3.4) in those without prior surgery. By the eighth year, the difference between the two groups had widened to 7.3% (95% CI 5.6 to 9.5) and 3.6% (95% CI 2.9 to 4.6), respectively. The overall discrepancy between the two cohorts was found to be statistically significant (p = 0.0026). The number at risk declined similarly in both groups over time, reflecting comparable follow-up patterns.

**Fig. 4:**
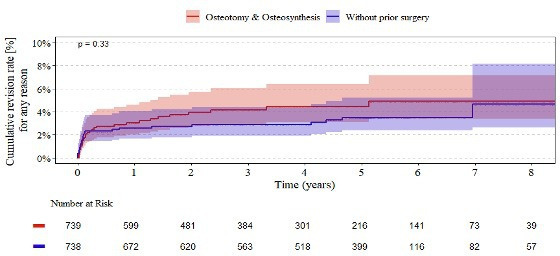
Endpoint revision of cemented femoral components. Kaplan–Meier curves showing eight-year revision rates for cemented femoral components in patients with (red curve) and without (blue curve) a history of osteotomy or osteosynthesis.

Figure 4 illustrates the Kaplan–Meier estimates for revision of cemented femoral components over eight years of follow-up. At one year, patients with prior osteotomy or osteosynthesis had a revision rate of 3.1% (95% CI 2.0 to 4.6), compared with 2.6% (95% CI 1.7 to 4.0) for those without prior surgery. By eight years, the cumulative revision rates were 4.9% (95% CI 3.4 to 7.1) and 4.7% (95% CI 2.6 to 8.2), respectively. The overall difference between the two groups was not statistically significant (p = 0.330). The number at risk decreased similarly across both cohorts throughout the observation period.

**Fig. 5:**
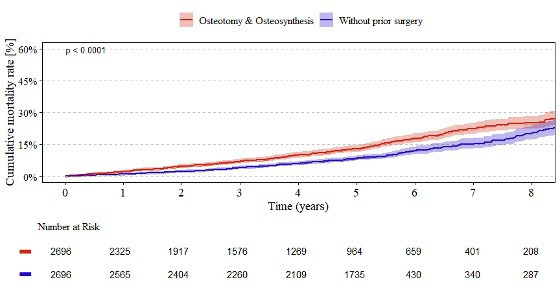
Endpoint mortality of all total hip arthroplasties. Kaplan–Meier estimates of the cumulative revision rate for total hip arthroplasty in patients with prior osteotomy or osteosynthesis compared to those without prior surgery, reported annually up to eight years postoperatively. Estimates are shown with their 95% CIs.

Figure 5 illustrates the cumulative mortality rates over an eight-year follow-up. At one year, the mortality estimate for patients with prior osteotomy or osteosynthesis was 2.2% (95% CI 1.7 to 2.8), compared to 1.0% (95% CI 0.7 to 1.5) in those without prior surgery. This difference increased steadily over time, reaching 25.2% (95% CI 22.5 to 28.3) compared with 20.4% (95% CI 17.4 to 23.8) at eight years. The overall between-group difference was statistically significant (p < 0.001). The number of patients at risk declined similarly in both cohorts, indicating comparable follow-up throughout the study period.

**Fig. 6:**
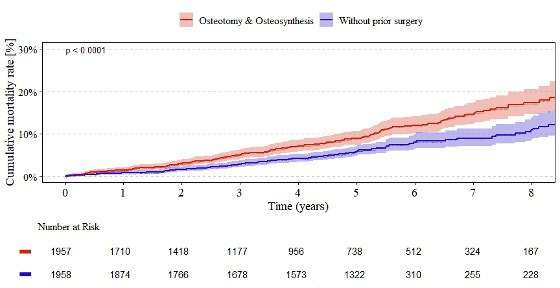
Endpoint mortality of uncemented femoral components. Kaplan–Meier curves showing the eight-year cumulative mortality in total hip arthroplasty patients with (red curve) and without (blue curve) a history of osteotomy or osteosynthesis.

Figure 6 presents the cumulative mortality rates for patients receiving uncemented femoral components. At one year, individuals with a history of osteotomy or osteosynthesis showed a mortality rate of 1.4% (95% CI 0.9 to 2.0), compared to 0.8% (95% CI 0.5 to 1.3) for those without prior surgery. This disparity in mortality rates widened progressively over time, reaching 17.3% (95% CI 14.5 to 20.6) in the osteotomy/osteosynthesis group compared to 10.9% (95% CI 8.6 to 13.8) in the control group after eight years. Overall, mortality was significantly higher in the osteotomy/osteosynthesis group (p < 0.001), and the number at risk declined similarly in both cohorts throughout the observation period.

**Fig. 7:**
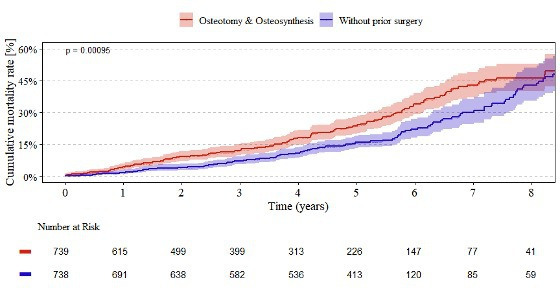
Endpoint mortality of cemented femoral components. Kaplan–Meier estimates of the eight-year cumulative mortality for cemented femoral components in patients with (red curve) and without (blue curve) a history of osteotomy or osteosynthesis, including 95% CIs.

Figure 7 presents the cumulative mortality rates for cemented femoral components over an eight‐year follow‐up. At one year, the mortality estimate among patients with prior osteotomy or osteosynthesis was 4.3% (95% CI 3.0 to 6.1), compared to 1.7% (95% CI 0.9 to 2.9) in those without such a history. This gap continued to expand throughout the study period, reaching 46.3% (95% CI 40.1 to 53.0) compared with 43.0% (95% CI 35.6 to 51.1) at eight years. The overall difference was statistically significant (p < 0.001). The two cohorts demonstrated analogous trends in declining numbers at risk, suggesting comparable follow‐up between them.

## Discussion

These findings, derived from a large, 1:1-matched cohort (n = 5,392), provide evidence of the impact that previous osteotomies or osteosyntheses can have on long-term outcomes following THA. Specifically, patients with such a history displayed an increased risk of revision and higher mortality rates. Notably, an elevated revision rate of 3.7% was observed in the first postoperative year, compared with 2.6% in the control group without surgical interventions. This disparity grew over time, culminating in rates of 6.8% compared with 3.9% after eight years (p = 0.002). Patients with previous operations exhibited a markedly higher mortality risk, rising from 2.2% to 25.2% between the first and eighth years, compared with 1.0% and 20.4% in the control cohort (p < 0.001).

A more nuanced examination of femoral component fixation methods (cemented vs cementless) supports these observations. A significant association between surgery and higher revision rates was detected among cementless femoral components (p = 0.003), but was less pronounced in cemented femoral components (p = 0.330). These results suggest that technical and biomechanical challenges—particularly pre-existing bony changes following osteotomies or osteosyntheses—may be more prevalent with cementless femoral components. Infections, periprosthetic fractures, and loosening of the cup or femoral component were the most common reasons for reoperation in both groups. Cup loosening (7.6% vs 5.3%) and femoral component loosening (6.9% vs 4.3%) occurred more frequently in the cohort with surgeries.

These findings may carry important implications for clinical practice. Our observations underscore that the anatomical and biomechanical alterations following osteotomies or osteosyntheses persist for many years, rendering patients more susceptible to implant failure and systemic complications. A notable strength of our study is the long-term data, which offer a more comprehensive perspective than studies that primarily focused on immediate or mid-term outcomes. The use of a 1:1 matching approach lends robustness to our conclusions by reducing confounding factors and facilitating a more reliable comparison with patients who had no prior interventions.

With regard to fixation methods, the higher revision rates among patients with cementless femoral components highlight the importance of thorough preoperative assessment. In contrast, cemented femoral components appeared less vulnerable, potentially because the cement mantle can compensate for structural irregularities and uneven bone quality. These findings underscore the need for strategies that integrate the patient’s surgical history and detailed imaging into the preoperative decision-making process.

Clinically, this study emphasizes the necessity of heightened vigilance regarding complications such as infections, periprosthetic fractures, and component loosening—issues that arise in structurally compromised bone. In particular, the increased incidence of cup or femoral component loosening among patients with a history of femoral or acetabular surgery highlights the importance of precise implant positioning and robust fixation. The marked increase in mortality rates suggests that the structural challenges and higher surgical complexity inherent to previously operated bone may play a pivotal role, reinforcing the need for a multidisciplinary approach from perioperative optimization to postoperative care.

Furthermore, post-traumatic cases—where fractures were initially treated by osteosynthesis—deserve attention, as multiple studies indicate that patients with such a background face a disproportionately higher risk of adverse outcomes. Similarly, Smith et al^14^ found that those undergoing THA after failed intertrochanteric fracture fixation with a cephalomedullary nail had higher complication rates and poorer outcomes compared to those without such a history. In addition to altered anatomy and potential bone loss, these individuals often exhibit higher infection rates and poorer overall health, contributing to both increased revision risk and elevated mortality.

The increased risks in this patient group can be traced back to several mechanisms. Drawing on recent studies, such as Gallazzi et al,^13^ Goh et al,^15^ and Duncan et al,^16^ four key clinical aspects emerge. First, following osteotomies (e.g. periacetabular or rotational), there may be localized reductions in bone density, leading to altered load distribution and increased risk of implant loosening and periprosthetic fractures.^13,15,16^ Second, biomechanical changes from an altered joint geometry or a shifted hip centre impose additional stress on a prosthesis, generating increased shear and rotational forces that elevate failure risk—especially with cementless press-fit femoral components.^17-19^ Third, scar tissue, deformities, and reduced bone stock complicate precise positioning and stable anchoring. Our data show that these anatomically complex conditions more frequently result in infections or suboptimal positioning.^20,21^ Fourth, compromised tissue or remnant osteosynthesis material predisposes patients to bacterial colonization, while scar-related changes and remodelling further impede proper prosthetic anchoring, raising infection risk in the long term.^16,18,19^

Taken together, these factors explain why patients with a history of osteotomy or osteosynthesis have significantly higher risks of revisions and mortality after THA. Meticulous evaluation of bone status, earliest and potentially staged removal of any osteosynthesis material, and selected fixation—particularly considering hybrid or cemented anchoring in compromised bone—are essential to reduce loosening, infection, and other complications. Our findings emphasize the importance of an interdisciplinary approach that includes infectious disease specialists, anaesthesiologists, and physical therapists to improve long-term care in this high-risk group. Future research should focus on refining surgical techniques and implant designs to reduce the high complication rates in compromised bone and altered hip anatomy.

Our subgroup analysis shows that cementless femoral components, in particular, are linked to a significantly higher revision risk in patients with a history of osteotomy or osteosynthesis (eight-year rate: 7.3% vs 3.6%, p = 0.003). Similar findings are reported by Crnogaca et al,^21^ who observed increased complications and revisions in patients with corrective osteotomies. Erdoğan and Can^18^ highlighted that scar tissue and poor bone quality from earlier surgeries adversely affect press-fit stability. In contrast, cemented femoral components appear more stable, as they showed comparable revision rates (4.9% vs 4.7%, p = 0.330). These findings align with Duncan et al,^16^ who similarly emphasized the benefits of cemented components in complex cases.

A comparison of our results with existing literature reveals both consistencies and differences. Hüsken et al^22^ analyzed patients with painful hip dysplasia who had undergone pelvic osteotomy and also reported increased risks of poorer outcomes. However, whereas they primarily identified ongoing dysplasia-related stress as the cause of reinterventions, we found that infections (17%) were the most common cause of revisions. This difference may be due to variations in surgical technique or postoperative care. Although Hüsken et al^22^ did not discuss significant differences between different fixation methods in the context of subsequent prosthesis implantation, our results highlight the superiority of cemented femoral component fixation in this high-risk group.

Compared to Ollivier et al,^23^ who studied a small cohort of patients with high-grade hip dysplasia (Crowe IV), our study shows significantly poorer outcomes in a larger and more heterogeneous population. Ollivier et al^23^ reported high ten-year implant survival rates and a marked improvement in clinical scores; however, these findings reflect specific patient data and are not directly transferable to our more heterogeneous cohort. The findings of this study underscore the challenges associated with the generalization of such results to complex patient populations.

Gallazzi et al^13^ investigated the use of different surgical approaches for THA and emphasized the importance of careful, stepwise removal of osteosynthesis material to minimize intraoperative risks. Although our study did not specifically examine this aspect, the comparatively high infection rate in our cohort suggests that adequate planning and consistent material removal are essential steps in risk reduction.

Park et al^24^ showed that patients with a history of pelvic osteotomies had a higher revision rate and poorer outcomes, especially when there were preoperative infections. Our analysis supports this observation, as infections represent a major risk factor in this patient group. By contrast, a systematic review by Duncan et al^16^ reported comparable survival and complication rates in patients with and without prior osteotomy. However, these findings were based on heterogeneous cohorts, while our matching approach permitted a more precise analysis.

Our results underscore the need for specific clinical strategies for these high-risk patients. Early evaluation of bone integrity and the choice of femoral component fixation method is crucial. Hybrid fixations should be preferred, especially in the presence of compromised bone stock, as they offer improved anchoring stability. Strict aseptic techniques and extended prophylactic measures could further reduce the risk of infection. In addition, multidisciplinary approaches are required to enhance postoperative care and rehabilitation.

The present study has several limitations. The EPRD collects extensive data from hospitals and health insurance providers, enabling consistent documentation of mortality and THA failure. However, the coding of diagnoses for comorbidities and postoperative complications is susceptible to human error and bias, a common issue in registries, which should be considered during data analysis. Furthermore, post-hospitalization data are limited to insurance records, meaning that not all complications could be captured. These limitations may affect the interpretability of our findings and should be taken into account when evaluating the study results.

In conclusion, these findings demonstrate that patients with a history of osteotomy or osteosynthesis have an increased risk of revision and mortality following THA. In summary, the data indicate that those with prior osteotomies or osteosyntheses face significantly higher revision and mortality rates, particularly when cementless femoral component fixation is used. Cemented femoral components appear to be a more promising option but still require careful surgical evaluation and precise intraoperative technique. Altogether, these results provide an important basis for improving treatment strategies and guiding future research in this complex patient population.

**Take home message**

- This study demonstrates that patients with a history of osteotomy or osteosynthesis prior to total hip arthroplasty face significantly higher long-term risks of revision and mortality compared to those without such a history.

- The findings highlight that cementless stem fixation is particularly associated with increased complications, underscoring the importance of careful surgical planning and fixation method selection in this high-risk group.

## Data Availability

The data that support the findings for this study are available to other researchers from the corresponding author upon reasonable request.

## References

[1] Parilla FW Freiman S Pashos GE Thapa S Clohisy JC Comparison of modern periacetabular osteotomy for hip dysplasia with total hip arthroplasty for hip osteoarthritis-10-year outcomes are comparable in young adult patients J Hip Preserv Surg 2022 9 3 178 184 10.1093/jhps/hnac029 35992023 PMC9389914

[2] Rollmann MF Holstein JH Pohlemann T et al. Predictors for secondary hip osteoarthritis after acetabular fractures-a pelvic registry study Int Orthop 2019 43 9 2167 2173 10.1007/s00264-018-4169-3 30267245

[3] Hoaglund FT Steinbach LS Primary osteoarthritis of the hip: etiology and epidemiology J Am Acad Orthop Surg 2001 9 5 320 327 10.5435/00124635-200109000-00005 11575911

[4] Jacobsen S Sonne-Holm S Hip dysplasia: a significant risk factor for the development of hip osteoarthritis. A cross-sectional survey Rheumatology (Oxford) 2005 44 2 211 218 10.1093/rheumtology/keh436 15479751

[5] Johnsen SP Sørensen HT Lucht U Søballe K Overgaard S Pedersen AB Patient-related predictors of implant failure after primary total hip replacement in the initial, short- and long-terms. A nationwide Danish follow-up study including 36,984 patients J Bone Joint Surg Br 2006 88-B 10 1303 1308 10.1302/0301-620X.88B10.17399 17012418

[6] Sanchez-Sotelo J Berry DJ Trousdale RT Cabanela ME Surgical treatment of developmental dysplasia of the hip in adults: II. Arthroplasty options J Am Acad Orthop Surg 2002 10 5 334 344 10.5435/00124635-200209000-00005 12374484

[7] Thillemann TM Pedersen AB Johnsen SP Søballe K Danish Hip Arthroplasty Registry Implant survival after primary total hip arthroplasty due to childhood hip disorders: results from the Danish Hip Arthroplasty Registry Acta Orthop 2008 79 6 769 776 10.1080/17453670810016830 19085493

[8] Jacobsen KK Laborie LB Kristiansen H et al. Genetics of hip dysplasia - a systematic literature review BMC Musculoskelet Disord 2024 25 1 762 10.1186/s12891-024-07795-2 39354451 PMC11445845

[9] Ganz R Leunig M Leunig-Ganz K Harris WH The etiology of osteoarthritis of the hip: an integrated mechanical concept Clin Orthop Relat Res 2008 466 2 264 272 10.1007/s11999-007-0060-z 18196405 PMC2505145

[10] No authors listed Endoprothesenregister Deutschland (EPRD) 2024 https://www.eprd.de/de date last accessed 29 July 2025

[11] No authors listed Operationen und Prozedurenschlussel (OPS-301), version 2025 Statistisches Bundesamt (Destatis), Deutsches Institut fur Medizinische Dokumentation und Information (DIMDI) 2024 https://www.bfarm.de/EN/Code-systems/Classifications/OPS-ICHI/OPS/_node.html date last accessed 4 August 2025

[12] World Health Organization International Statistical Classification of Diseases and Related Health Problems, 10th Revision (ICD-10) https://icd.who.int/ date last accessed 1 August 2025

[13] Gallazzi E Morelli I Peretti G Zagra L What is the impact of a previous femoral osteotomy on THA? A systematic review Clin Orthop Relat Res 2019 477 5 1176 1187 10.1097/CORR.0000000000000659 30998636 PMC6494317

[14] Smith A Denehy K Ong KL Lau E Hagan D Malkani A Total hip arthroplasty following failed intertrochanteric hip fracture fixation treated with a cephalomedullary nail Bone Joint J 2019 101-B 6_Supple_B 91 96 10.1302/0301-620X.101B6.BJJ-2018-1375.R1 31146562

[15] Goh EL Boughton OR Donnelly T Murphy CG Cashman J Green C Do joint-preserving hip procedures compromise subsequent total hip arthroplasty? A meta-analysis of complications, functional outcome and survivorship SICOT J 2024 10 25 10.1051/sicotj/2024018 38847649 PMC11160402

[16] Duncan S Wingerter S Keith A Fowler SA Clohisy J Does previous osteotomy compromise total hip arthroplasty? A systematic review J Arthroplasty 2015 30 1 79 85 10.1016/j.arth.2014.08.030 25262440

[17] Yacovelli S Abdelaal M Fillingham Y Sutton R Madding R Parvizi J Prior pelvic osteotomy affects the outcome of subsequent total hip arthroplasty J Arthroplasty 2021 36 2 600 604 10.1016/j.arth.2020.07.080 32917462

[18] Erdoğan F Can A The effect of previous pelvic or proximal femoral osteotomy on the outcomes of total hip arthroplasty in patients with dysplastic coxarthrosis Acta Orthop Traumatol Turc 2020 54 1 74 82 10.5152/j.aott.2020.01.7 32175900 PMC7243695

[19] Esmaeili S Ghaseminejad-Raeini A Ghane G Soleimani M Mortazavi SMJ Shafiei SH Total hip arthroplasty in patients who have crowe type IV developmental dysplasia of the hip: a systematic review J Arthroplasty 2024 39 10 2645 2660 10.1016/j.arth.2024.05.031 38759817

[20] Fahlbusch H Budin M Volk A et al. Long-term outcomes of total hip arthroplasty in patients with developmental dysplasia of the hip: a minimum 21-year follow-up Arch Orthop Trauma Surg 2023 143 11 6609 6616 10.1007/s00402-023-04970-3 37421515

[21] Crnogaca K Sulje Z Delimar D Previous corrective osteotomies of femur and pelvis are a risk factor for complications following total hip arthroplasty in hip dysplasia J Orthop 2022 33 100 104 10.1016/j.jor.2022.07.008 35942332 PMC9356201

[22] Hüsken MFT Magré J Willemsen K et al. Association of osteotomy, age, and component fixation with the outcomes of total hip arthroplasty in patients with hip dysplasia: a Dutch population-based registry study Acta Orthop 2024 95 545 552 10.2340/17453674.2024.41383 39269264 PMC11395819

[23] Ollivier M Abdel MP Krych AJ Trousdale RT Berry DJ Long-term results of total hip arthroplasty with shortening subtrochanteric osteotomy in crowe IV developmental dysplasia J Arthroplasty 2016 31 8 1756 1760 10.1016/j.arth.2016.01.049 26952206

[24] Park CW Lim SJ Cha YT Park YS Total hip arthroplasty with subtrochanteric shortening osteotomy in patients with high hip dislocation secondary to childhood septic arthritis: a matched comparative study with crowe IV developmental dysplasia J Arthroplasty 2020 35 1 204 211 10.1016/j.arth.2019.08.034 31521447

